# Being Flexible: The Voltage-Controllable Activation Gate of Kv Channels

**DOI:** 10.3389/fphar.2012.00168

**Published:** 2012-09-13

**Authors:** Alain J. Labro, Dirk J. Snyders

**Affiliations:** ^1^Department of Biomedical Sciences, University of AntwerpAntwerp, Belgium

**Keywords:** bundle crossing gate, glycine and PXP hinge point, pore opening and closure, selectivity filter, shaker potassium channel, voltage-dependent gating

## Abstract

Kv channels form voltage-dependent potassium selective pores in the outer cell membrane and are composed out of four α-subunits, each having six membrane-spanning α-helices (S1–S6). The α-subunits tetramerize such that the S5–S6 pore domains co-assemble into a centrally located K^+^ pore which is surrounded by four operational voltage-sensing domains (VSD) that are each formed by the S1–S4 segments. Consequently, each subunit is capable of responding to changes in membrane potential and dictates whether the pore should be conductive or not. K^+^ permeation through the pore can be sealed off by two separate gates in series: (a) at the inner S6 bundle crossing (BC gate) and (b) at the level of the selectivity filter (SF gate) located at the extracellular entrance of the pore. Within the last years a general consensus emerged that a direct communication between the S4S5-linker and the bottom part of S6 (S6_c_) constitutes the coupling with the VSD thus making the BC gate the main voltage-controllable activation gate. While the BC gate listens to the VSD, the SF changes its conformation depending on the status of the BC gate. Through the eyes of an entering K^+^ ion, the operation of the BC gate apparatus can be compared with the iris-like motion of the diaphragm from a camera whereby its diameter widens. Two main gating motions have been proposed to create this BC gate widening: (1) tilting of the helix whereby the S6 converts from a straight α-helix to a tilted one or (2) swiveling of the S6_c_ whereby the S6 remains bent. Such motions require a flexible hinge that decouples the pre- and post-hinge segment. Roughly at the middle of the S6 there exists a highly conserved glycine residue and a tandem proline motif that seem to fulfill the role of a gating hinge which allows for tilting/swiveling/rotations of the post-hinge S6 segment. In this review we delineate our current view on the operation of the BC gate for controlling K^+^ permeation in Kv channels.

## Introduction

Potassium (K) channels form transmembrane permeation pathways (pores) with a high selectivity for K^+^ over other monovalent ions like Na^+^. *In vivo*, these channels are responsible for repolarizing the membrane potential back to its resting condition following an action potential, to set the resting membrane potential of the cell and to determine the action potential firing rate (Hille, 2001). The biophysical properties and abundance of these channels shape the time course of the action potential, and constitute a critical determining factor of cellular excitability. To serve their *in vivo* role, the flow of K^+^ needs to be strictly controlled and channels need to be able to actively open or close their pore in response to varying stimuli such as changes in pH or Ca^2+^/ligand concentration. In the case of voltage-gated potassium (Kv) channels, which are the predominant K channels shaping the action potential duration, this stimulus is a change in membrane potential.

**Figure 1:**
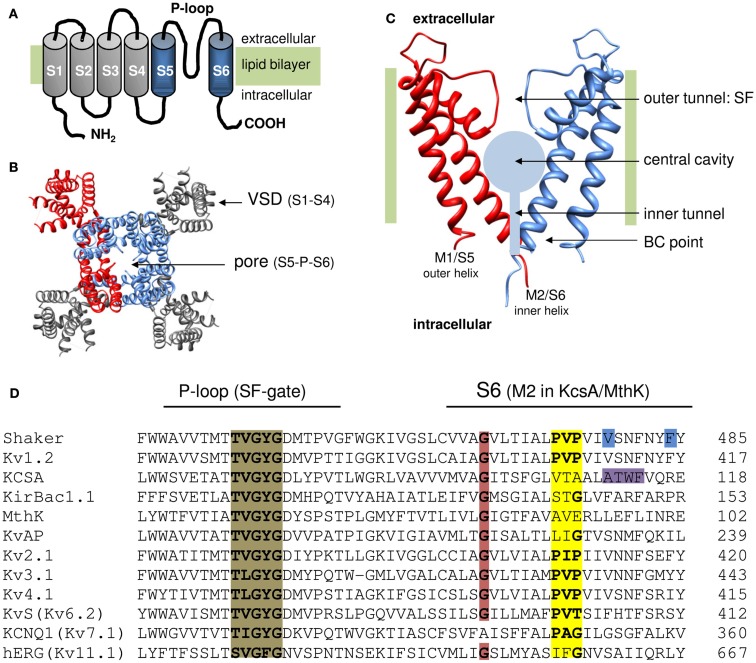
**Topology of K channels**. **(A)** Cartoon of the six transmembrane segment (S1–S6) one P-loop (6Tm-1P) topology of a Kv channel α-subunit with both amino (NH_2_) and carboxyl (COOH) terminus located intracellular. The S1–S4 segments form the VSD (represented in gray) and the S5-P-loop-S6 region assembles with three other pore domains into the K^+^ permeation pathway. **(B)** Top view (from the extracellular side) of the 3D structure of the Kv1.2 channel (protein data bank accession code 2A79; Long et al., 2005). To illustrate the fourfold symmetrical assembly of the α-subunits into a functional channel, one α-subunit is represented in red. In the other subunits the pore region (S5-P-loop-S6) is colored blue and the VSD (S1–S4) is represented in gray. Note that the pore regions form a centrally located K^+^ pore that is surrounded by four independent VSDs. **(C)** Side view of the pore module of the 2Tm-1P K channel KcsA that was crystallized in the closed state (protein data bank accession code 1BL8; Doyle et al., 1998). The first transmembrane segment M1 (which resembles S5 in Kv channels) locates at the periphery and faces the lipid bilayer whereas the second transmembrane segment M2 (corresponding to S6) forms the inner pore helix. The front and back α-subunit are omitted to illustrate the layout of the K^+^ permeation pathway that – from the intracellular to the extracellular side – can be divided in three recognizable sections; (1) a water filled inner tunnel, (2) a wider 12 Å in diameter water filled cavity, and (3) a narrower outer tunnel that forms the ion selectivity filter (SF) that dictates K^+^ selectivity. Both the inner tunnel and the central cavity are formed by the inner pore helices that cross the membrane under an angle of ∼25° making the resemblance with an inverted teepee (Doyle et al., 1998). The K^+^ pathway contains two energy barriers for K^+^ that function as a gate: (1) at the bundle crossing (BC) of the M2/S6 helices (BC gate) that forms a barrier for hydrated K^+^ and (2) the SF that allows passage of K^+^ ions which have shed their hydration shell. **(D)** Sequence alignment of the inner pore helix of the pore module (S6 and M2 segment, respectively), and the P-loop that forms the channels SF which contains the TVGYGD signature sequence (highlighted in brown) for a K^+^ selective channel (Heginbotham et al., 1994). Highlighted in red is the highly conserved glycine residue in the middle of the inner pore helix. The PXP motif present in Kv channels is highlighted in yellow. Note that in the “silent” Kv channels (KvS, with the Kv6.2 member represented) the second proline of the PXP motif is lacking. The residues proposed to seal off the K^+^ pore in *Shaker* (gate residues V478 and F484) are highlighted in blue and the residues at the level of the BC in KcsA are highlighted in purple.

A typical Kv channel is composed of four individual α-subunits (MacKinnon, 1991), each containing six membrane spanning helices (S1–S6) organized to form a central K^+^ pore with the S5 and S6 segments (Figures 1A,B; Doyle et al., 1998; Long et al., 2005). The S4 segment is positively charged and assembles with the S1–S3 segments into a voltage-sensing domain (VSD) that detects changes in membrane potential. Since each subunit has its own VSD, a functional channel consists out of one centrally located K^+^ pore that is surrounded by four operational VSDs. Membrane re- or depolarization creates a force on the VSD causing its movement. This molecular rearrangement is transmitted via an electromechanical coupling to the channel’s activation gate(s) that seals off the K^+^ pore. K^+^ permeation can be sealed off by two separate gates in series: (a) at the inner S6 bundle crossing (BC; Liu et al., 1997; del Camino and Yellen, 2001) and (b) at the level of the selectivity filter (SF; Liu et al., 1996; Loots and Isacoff, 1998; Cuello et al., 2010b). An in depth review on the operation of the VSD and electromechanical coupling has been given by others in this research topic of Frontiers in Pharmacology (Blunck and Batulan, 2012; Delemotte et al., 2012; Vardanyan and Pongs, 2012). Here we delineate the current view on the operation of the channel’s activation gate for which most of our understanding comes from studies in the prototypical *Shaker* Kv channel. Therefore the detailed findings and residue numbering are from *Shaker* unless mentioned otherwise.

## Location of the Bundle Crossing Gate

The first evidence for the presence of a voltage-controllable gate that seals off K^+^ permeation at the intracellular entrance of the channel pore came from blocking experiments in giant squid axons using quaternary ammonium (QA) derivatives such as tetraethylammonium (TEA). These seminal studies showed that intracellularly applied QA derivatives blocked the K^+^ current only after opening of the channels (Armstrong, 1966, 1971; Armstrong and Hille, 1972). Furthermore, when the QA derivatives were bound and induced current block, they impeded the closure of the intracellular gate during membrane repolarization making the resemblance with a foot in the door mechanism. About 20 years later the first *Shaker* Kv channel was cloned (Papazian et al., 1987; Timpe et al., 1988), and the drug blocking experiments were repeated yielding similar results (K^+^ permeation through these *Shaker* channels behaved like the K^+^ currents in giant squid axons) strengthening the hypothesis of a gate at the intracellular entrance of the K^+^ pore (Choi et al., 1993). With a growing number of cloned Kv channels and improved molecular biology techniques, structure-function mutagenesis studies indicated that residues within the S6 transmembrane segment affected the binding affinity for these QA derivatives (Hartmann et al., 1991; Yellen et al., 1991; Choi et al., 1993; Shieh and Kirsch, 1994; Taglialatela et al., 1994). These results provided the first evidence for the S6 segment being involved in lining the K^+^ permeation pathway and housing the intracellularly located activation gate.

The location of this intracellular S6 activation gate was determined by investigating the chemical modification rate of introduced cysteine residues by organic derivates (MTS reagents) and Cd^2+^ (Liu et al., 1997). Comparison of the state-dependent accessibility of several S6 residues (closed vs. open) revealed that the residues above I477 were only modified by MTS reagents or Cd^2+^ – and thus accessible – when the channel gate was open. Residues below I477 were always accessible and no difference was observed between a closed or open gate conformation. The fact that modification of all tested residues was prevented by larger QA derivatives indicated that these residues effectively line the K^+^ permeation pathway. From the different residues tested, V474C showed the highest difference in modification rate between the closed and open conformation (Liu et al., 1997; del Camino and Yellen, 2001). Although Cd^2+^ binding to V474C and channel block occurred irreversibly, the unblock could be monitored using reducing reagents (e.g., DTT) and appeared to be highly voltage-dependent suggesting that residue V474 is located above the gate residue(s) that seal off the K^+^ pore. The observation that the Cd^2+^ block did not affect the voltage-dependent gating kinetics of the channel led the authors to propose, based on testing different gating schemes, that the position of the cysteine does not change much between the closed and open gate conformation. This supported the hypothesis that the voltage-dependent accessibility to this residue was not because it lies buried within the channel protein in the closed state but because it effectively lines the K^+^ permeation pathway and access to it is controlled by the intracellular S6 activation gate below this level (Liu et al., 1997; Webster et al., 2004).

Since the access of Cd^2+^, that is smaller in size than K^+^, was well controlled by this intracellular S6 activation gate it was conceivable that also K^+^ would be retained. However, Cd^2+^ is a divalent and has a stronger hydration shell than K^+^ making it not a perfect substitute. Repeating the accessibility studies with the monovalent silver Ag^+^ ion (that serves as a better substitute for K^+^ than Cd^2+^; Lue and Miller, 1995) showed a 700-fold difference in the modification rate of V474C between the closed and open channel conformation. These data showed that the K^+^ flow can be sealed off at the level of his intracellular S6 activation gate (del Camino and Yellen, 2001). However, since also the access of much bigger QA derivatives is controlled, this intracellular S6 activation gate is most likely not involved in determining K^+^ selectivity and its sole role would be to control the K^+^ flow.

## The Intracellular Channel Gate Works as a Hydrophobic Seal

The studies detailed above strongly supported the notion for the presence of an activation gate for K^+^ ions in the bottom part of the S6 segment (around or below residue I477) at the intracellular entrance of the channel pore (Liu et al., 1997). In an extensive mutagenesis scan of the S6 to pinpoint the gate residue, all residues were mutated to either a tryptophan or alanine (Hackos et al., 2002). Tryptophan was used to increase side-chain volume substantially as to narrow the gate and promote the closed state. On the contrary, an alanine would reduce side-chain volume and might increase the gate radius which would promote channel opening. The scan revealed residues V478 and F484 as strong candidates to be the effective gate residue(s) (Figure 1D; Hackos et al., 2002). In agreement, substituting P475 by a charged aspartate (that results in charge-charge repulsion and thus promotes gate widening) resulted in a constitutive conducting K^+^ pore (Sukhareva et al., 2003). Mutating F484 to a cysteine reduced not only the open probability but also the single-channel conductance supporting that this residue forms an energy barrier for electrodiffusion of K^+^ ions (Ding and Horn, 2002). The fact that both candidates for being the gate residue(s) were not charged or strongly polarized, favored the idea that the nature of the gate relied on creating a hydrophobic barrier for K^+^ ions instead of an electrostatic field effect (del Camino and Yellen, 2001). Accordingly, the channel gate could be trapped in the closed conformation by a tryptophan substitution for residue V478 that resulted in the formation of a hydrophobic seal, strengthening the idea that the gate residue(s) form(s) a steric hindrance for K^+^ permeation (Kitaguchi et al., 2004). Energy calculations indicated that with a strong constriction and small pore radii (<2 Å) the energy barrier for K^+^ to pass indeed originates from Van der Waals interactions (i.e., a hydrophobic seal; Tai et al., 2009).

The elucidation of the 3D crystal structure of the two transmembrane one pore (2Tm-1P) K channel KcsA, a prokaryotic K channel that is not gated by voltage, provided the first detailed picture of a channel pore and greatly advanced our understanding of ion permeation (Doyle et al., 1998). The two transmembrane segments (M1 and M2) of KcsA correspond to the S5 and S6 segments of the *Shaker* Kv channel and could even substitute them (Lu et al., 2001). The initial suggestion that the KcsA structure represents a well conserved outline of a K^+^ pore found in many types of K channels has been confirmed by the crystallization of other K channels (Jiang et al., 2002a; Tao et al., 2009) including the voltage-gated bacterial KvAP channel (Jiang et al., 2003) and more recently the *Shaker*-related Kv1.2 channel (Long et al., 2005, 2007). The pore, formed by either the M1–M2 or S5–S6 segments and the P-loop between them, shows a fourfold symmetry whereby the individual α-subunits arrange themselves around the ion-conducting pathway that can be divided in three regions. From the intracellular to the extracellular side, the pathway starts with (1) a water filled tunnel which opens into (2) a wider water filled cavity. Both these regions are delineated by the four inner hydrophobic pore helices (the M2 or S6 segments) that cross the membrane under an angle of ∼25° (Figure 1C). At the end of the cavity starts (3) a second narrower outer tunnel that is formed by the P-loops and forms the channel’s SF that dictates K^+^ selectivity.

Since the M2 helices in KcsA traverse the plasma membrane under an angle of ∼25° and cross each other at the intracellular entrance of the pore, the resemblance was made with the supporting rods of an inverted teepee (Doyle et al., 1998). At the BC point of the four helices the channel pore is constricted such that it would be impossible for K^+^ to pass, indicating that the initial KcsA structure represented the closed conformation. Based on sequence alignment (Figure 1D), the residues that appeared in *Shaker* to be accessible only in the open channel conformation are indeed located above this BC point. Therefore, this BC of the M2 (S6) helices most likely forms the intracellular activation or BC gate. As previously suggested, this BC gate controls the K^+^ flow but does not dictate K^+^ selectivity which is limited to the SF.

Later, another 2Tm-1P prokaryotic K channel (KirBac1.1) was crystallized in the closed state showing a similar pore architecture as KcsA (Kuo et al., 2003): the M2 helices formed a BC gate and a central cavity that could house hydrated K^+^ ions lowering the energetic cost for putting a charged ion in the middle of the lipid bilayer (Doyle et al., 1998; Roux and MacKinnon, 1999). Both structures suggested that closure of the BC gate does not involve a large scale collapse of the permeation pore but is limited to a pore constriction at the level of the BC point. This is further evidenced by the observation that QA derivatives could be trapped behind the intracellular activation (BC) gate upon channel closure in the *Shaker* pore mutant I470C (Holmgren et al., 1997). This indicated that in the I470C mutant there is sufficient room behind the BC gate to house QA derivatives up to a diameter of 8–10 Å. Bigger derivatives do not fit completely and by sticking out they function as a foot in the door keeping the BC gate open upon membrane repolarization that normally promotes channel closure. In the hERG (Kv11.1) Kv channel the space behind the BC gate is even larger than in *Shaker* as also bigger components could be trapped (Mitcheson et al., 2000). Consequently, the BC gate acts as a trap door mechanism for QA derivatives and small blockers such that they are retained within the central cavity behind the closed BC gate (Holmgren et al., 1997; Liu et al., 1997). The crystallization of KcsA in the presence of tetrabutylammonium and subsequent structure determination showed that QA derivates can indeed occupy the central cavity when the channel is closed (Zhou et al., 2001).

Such a trap door mechanism for BC gate operation strengthens the concept that channel closure does not involve mayor rearrangements of the permeation pathway, i.e., a full collapse of the central cavity. However, recent MD simulation studies pointed to the role of hydrophobic changes and dewetting (water leaving) of the pore and cavity for controlling the K^+^ flow in Kv channels (Jensen et al., 2010, 2012). Previous simulations on simple nanopores showed that a hydrophobic pore with a diameter of 6 Å forms already a considerable energy barrier for water and consequently hydrated K^+^ ions. This indicates that the BC gate does not need to physically occlude completely to a diameter smaller than the size of a K^+^ ion (2.66 Å diameter) in order to be functionally closed and shut off the K^+^ flow (Beckstein et al., 2003, 2004). In the recently proposed hydrophobic gating mechanism for Kv channels, BC gate closure is preceded by dewetting the pore which is sufficient to terminate the K^+^ flow. Conversely, BC gate opening proceeds after rehydration of the pore. These dewetting and rehydration processes involve effective rearrangements of the pore and a partial collapse of the central cavity (Jensen et al., 2010, 2012). Therefore, the hypothesis that the cavity is structurally different between the closed and the open channel state is quite conceivable but a full collapse seems unlikely as it would be inconsistent with the trapping of QA derivatives and other drugs behind the BC gate.

## BC Gate Movement is Under Control of the VSD

The BC gate appears to comprise the V478 and/or F484 residues that seal off the permeation pathway by forming a hydrophobic constriction (Figure 1D). The observation that (a) accessibility of an introduced cysteine at residue position 474, located above this hydrophobic seal, followed the voltage-dependence of channel opening and (b) mutations in the BC gate region affected the VSD movement (Ding and Horn, 2003) strongly indicated that the BC gate communicates directly with the VSD and forms the channel’s main voltage-controllable activation gate.

The conductance for K^+^ in the closed state was estimated to be about 100,000 times lower than that of the open state showing tight closure of the ion pore (Soler-Llavina et al., 2003). It has been well established that the channel traverses multiple closed states before reaching the “activated-not-open” state followed by a final transition that results in BC gate opening (Bezanilla et al., 1994; Zagotta et al., 1994; Schoppa and Sigworth, 1998; Zandany et al., 2008). Most of these states reflect different VSD conformations but also the status of the BC gate is different between the activated and the open state (del Camino et al., 2005). This raises the question whether conformational changes of the BC gate in the pre-open states are sufficient to allow ion permeation and result in different conducting states. Due to its fourfold symmetry each channel has four S6 gate regions that each communicate with their own VSD (Labro et al., 2005). For *Shaker* it is generally assumed that the four S6 gate regions operate in a cooperative manner and that BC gate opening occurs in a concerted step (Zandany et al., 2008) when all four VSDs have reached their activated state. However, this does not need to be a universal rule and pre-open BC gate movements might be sufficient to allow passage of K^+^ ions in certain K channels. In the case of the Kv2.1 channel (previously named drk1), that has a higher single-channel conductance than *Shaker*, subconductance levels for K^+^ have indeed been observed in the early steps of channel opening and during channel closure (Chapman et al., 1997; Chapman and VanDongen, 2005). This suggests that, at least for Kv2.1, the movement of one S6 helix is sufficient to have partial pore opening which results in subconductance levels. Accordingly, occupancy of the first subconductance state was linked to the movement of the first of the four VSDs (Chapman and VanDongen, 2005).

Possibly, these independent S6 movements can only be observed directly as subconductance states in K^+^ channels with a relative high single-channel conductance: in channels with lower single-channel conductance the amplitude of the subconductance states would be too low and hidden within the noise. Alternatively, a tight packing of the S6 helices at the BC imposes a high cooperativity in the movement of the S6 helices and BC gate making the subconductance states too short lived to be detected (Gagnon and Bezanilla, 2010). In *Shaker* the BC gate constriction (S6 bundle packing) in the closed state is probably very tight and BC gate opening occurs in a concerted step that could be isolated by introduction of the ILT mutations in the channel’s gating machinery (Ledwell and Aldrich, 1999). Using a mutant *Shaker* construct that displays an increased single-channel conductance and an open state stabilization, subconductance levels were observed during both channel activation and deactivation supporting that subconductance states also exist in *Shaker* but are too short lived or too small to be detected directly (Zheng and Sigworth, 1997, 1998). When the channel opens, the BC gate widens and the S6 BC becomes less packed. Using hidden Markov model analysis subconductance states could indeed be resolved during deactivation of Wild-Type *Shaker* channels (Zheng et al., 2001). Since these subconductance states reflect the behavior of individual subunits, this predicts that ionic current deactivation should follow VSD return. Detailed studies on the movement of the VSD of *Shaker* indeed revealed such a correlation and full BC gate closure (shutting off the K^+^ flow) occurred only when the last VSD had returned to its rested state (Bezanilla et al., 1991). Although these data suggest the presence of subconductance levels, shutting down the K^+^ flow is most likely not a gradual process that develops with each subunit moving as the largest decay in ionic current deactivation (more than 3/4 of the total amplitude) precedes full gating charge return (Varga et al., 2002). Recent MD modeling studies showed that closure of the S6 gate region from one subunit is sufficient to prevent almost any hydrated K^+^ from passing the BC gate indicating that the VSD return of one subunit causes the largest drop in K^+^ flow through the pore (Jensen et al., 2010, 2012).

## The BC Diameter as Potential Basis for Single-Channel Conductance and Subconductance Levels

**Figure 2:**
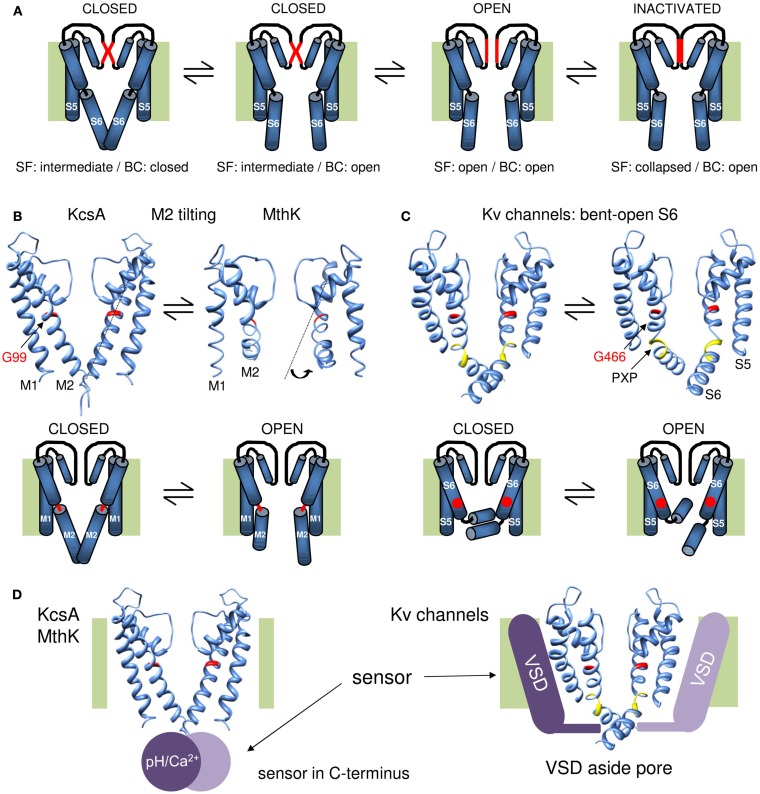
**Overview of the gating mechanisms for pore opening in K channels**. **(A)** Cartoon representation of a gating mechanism involving the sequential opening of two gates: the BC and SF gate. Assuming that the SF is in an intermediate conducting state in the closed channel conformation, the channel has both its SF and BC gate closed. Upon membrane depolarization (or other stimulus that triggers channel opening) the BC gate opens but the SF remains in its intermediate conducting state. This BC gate opening subsequently triggers the SF to open and results in full channel opening. Upon prolonged depolarization (or other stimulus) the SF collapses and the channel enters the inactivated state. **(B)** KcsA/MthK gating mechanism that involves conversion of a straight inner M2 pore helix (closed conformation) into a conformation whereby the M2 helix splays open at the level of a conserved glycine residue (G99 in KcsA). On top the 3D crystal structures of KcsA on the left (closed state) and MthK on the right (open conformation, protein data bank accession code 1LNQ; Doyle et al., 1998; Jiang et al., 2002a). Note the different conformation of the M2 helix that tilts at the level of a glycine residue in the middle of the helix (indicated in red). Below a cartoon representation of the proposed gating mechanism whereby the glycine forms a hinge point (indicated in red) and opening of the BC gate requires tilting of the post-hinge M2 segment. **(C)** Proposed gating mechanism for *Shaker*-type Kv channels. On the right the 3D crystal structure of the Kv1.2 channel in the open conformation and on the left a model for the closed state built by Pathak et al. (2007). Note that the inner S6 pore helix remains bent in both closed and open conformation resulting in the “bent-open S6 model” for channel gating. The glycine counterpart that forms the hinge in KcsA/MthK is G466 (indicated in red). However, the bend in S6 is not at this glycine residue but at the conserved PXP motif (colored yellow) located seven residues further downstream. Below a cartoon representation of the proposed gating mechanism whereby G466 is indicated with a red dot. In contrast to the mechanism in **(B)**, most of the reorientations in S6 occur in the vicinity of the PXP motif. **(D)** Illustration for the location of the stimulus sensor in KcsA/MthK vs. Kv channels. Left: the 3D structure of KcsA with the pH sensor (Ca^2+^ sensor in case of MthK) indicated with a purple sphere that locates in the C-terminus underneath the inner M2 pore helix. Right: the Kv1.2 structure with the VSD indicated with a purple bar situated besides/adjacent to the K^+^ pore. The different location of the stimulus sensor that controls the status of the BC gate may explain their different gating mechanism proposed in **(B,C)**.

The underlying structural basis for these subconductance levels in Kv2.1 channels has not been unequivocally determined but most likely they find their origin in individual S6 movements. However, since K^+^ conduction in the closed state is about 100,000 times lower than in the open one (Soler-Llavina et al., 2003) but Ag^+^ and Cd^2+^ modification of residue V474C displays only a 700-fold difference between both states (del Camino and Yellen, 2001), one may question whether the BC gate is solely responsible for controlling the K^+^ flow. Within the mechanism of hydrophobic pore gating (discussed above) these contradicting data could be reconciled if K^+^ flow is halted by dewetting the pore which does not require a fully closed BC gate (Jensen et al., 2012). On the other hand, it has been shown that the subconductance levels in the T442S *Shaker* chimera have a different ion selectivity suggesting that also the SF is involved (Zheng and Sigworth, 1997). Since S6 undergoes overall structural changes upon BC gate opening that are transmitted upward along the S6 helix and can trigger the closure of the SF during the process of C-type inactivation (Cuello et al., 2010a,b), it is possible that the conformation of the SF also differs when the BC gate is open or closed. In this speculative scenario the SF would have three main conformations; (1) the SF is in an intermediate conducting state when the BC gate is closed but converts to (2) a higher conducting state upon BC gate opening and finally (3) collapses during C-type inactivation (Figure 2A). The observed changes in ion selectivity are in support of this scenario that implies the presence of two activation gates. Fluorescence lifetime spectroscopy in KcsA channels showed indeed a discrepancy between BC gate opening and actual K^+^ conduction pointing to a role of the SF as a second gate (Blunck et al., 2006). Interestingly, in several 2Tm-1P K channels (Claydon et al., 2003; Clarke et al., 2010) and in 6Tm-1P cyclic nucleotide gated and Ca^2+^ gated eukaryotic K channels, K^+^ permeation seems to be fully controlled at the level of the SF (Sun et al., 1996; Flynn and Zagotta, 2001; Contreras et al., 2008; Thompson and Begenisich, 2012). Although in Kv channels K^+^ permeation appears to be mainly controlled by the BC gate, there is strong evidence for a direct communication between the BC gate and the SF (Panyi and Deutsch, 2006; Cuello et al., 2010a,b). Since in most Kv channels full BC gate opening develops during a final concerted step, intra-subunit pre-open S6 movements might already result in rearrangements of both the central cavity and the SF. It has been shown that the conformation of the BC gate (and consequently S6) in the pre-open state differs from the closed and the open one (del Camino et al., 2005). Furthermore, MD simulations show that subtle motions in the side chains of S6 residues can alter the behavior of the subunits and affect larger scale rearrangements (Denning and Woolf, 2010). Such a mechanism of pre-open S6 movements can form the basis for closed state inactivation in certain types of Kv channels (Barghaan and Bahring, 2009; Bahring and Covarrubias, 2011; Bahring et al., 2012) and might explain the modulating role of individual Kv α-subunits in a heterotetrameric configuration as in Kv6.4/Kv2.1 channels (Bocksteins et al., 2012).

Although the exact gate movements remain largely undefined, they must involve movement of the S6 helices relative to each other because a metal bridge between V476C and H486 in adjacent subunits can lock the BC gate in the open state (Holmgren et al., 1998). This strongly suggests that the opening and closure of the BC gate involves larger scale movements of the bottom part of the S6 (S6_c_) segment. This brings us to the question, does the diameter of BC gate opening determines the single-channel conductance? Residue substitutions in the BC gate vicinity indeed affected the single-channel conductance of Kv channels supporting that the BC gate forms an energy barrier that can affect the K^+^ flow rate (Lopez et al., 1994; Shieh and Kirsch, 1994; Ding and Horn, 2002). Substituting the S6 in *Shaker* by Kv3.1 sequence, which has a higher single-channel conductance, indeed resulted in a *Shaker* chimera that displayed an increased single-channel conductance compared to WT channels (Lopez et al., 1994; Taglialatela et al., 1994). Within this chimera the sequence of the SF was conserved and exchanging the SF region between *Shaker* and Kv3.1 had indeed no impact on the single-channel conductance (Taglialatela et al., 1994). Accordingly, the different types of K channels crystallized to date display a very similar SF structure although they have quite different single-channel conductances. Therefore, the SF is most likely not involved in tuning the conductance to this extent, but its configuration is mainly conserved and optimized to highly discriminate K^+^ over Na^+^ at a 1000:1 ratio and at the same time allowing K^+^ flow at a high rate (∼10^7^ ions s^−1^; Morais-Cabral et al., 2001). Although more experimental support is needed, it appears that the BC gate not only seals off the pore when closed but also controls the K^+^ flow rate when open (i.e., the single-channel conductance). Consequently, an alternative and more speculative explanation for the presence of subconductance levels is the existence of intermediate conducting BC gate conformations that originate from non-concerted S6 movements of individual subunits. Indeed, recent studies on the gating mechanism of Kv7.1 (KCNQ1) channels showed that BC gate opening in this type of Kv channels does not require that all four VSDs have moved to their activated state (Ma et al., 2011; Osteen et al., 2012). Although Kv7.1 may be an isolated case, such gating mechanism yields several “intermediate” open states of the BC gate that originate from individual VSD movements.

Although the SF may function as a second gate in series to the BC gate and may undergo conformational changes during activation, most likely the BC gate remains the main activation gate in Kv channels that is under tight control of the VSD. Over the past years a general consensus arose that in most Kv channels the electromechanical coupling that links the BC gate region with the VSD originates from a molecular communication between the S4S5-linker (S4S5_L_) and S6_c_ (see Blunck and Batulan, 2012; Vardanyan and Pongs, 2012 in this research topic of Frontiers in Pharmacology). Subsequently, these S6_c_ movements that lead to BC gate opening are also transmitted along the entire six helix up to the level of the SF that can respond by changing its conformation. The induced conformational change might destabilize the SF and trigger C-type inactivation that results in a full collapse of the SF when all the K^+^ ions have left the SF (Baukrowitz and Yellen, 1996; Ogielska and Aldrich, 1999). Consequently, altering the K^+^ concentration in the extracellular or intracellular milieu (affecting the K^+^ flow through the channel pore) affects the speed of C-type inactivation (Lopez-Barneo et al., 1993; Baukrowitz and Yellen, 1995). Interestingly, these K^+^-dependent effects on SF gating and C-type inactivation in general are reflected in the speed of BC gate opening and closure (Starkus et al., 1997; Ader et al., 2009). This indicates that the communication between both gates is mutual and that molecular motions within the SF (i.e., during C-type inactivation) may modulate the behavior of the BC gate which is controlled by the VSD.

## Movements of the BC Gate in the Transition from Closed to Open

The observation that the BC gate could be locked open by the formation of an inter-subunit Cd^2+^ bridge between an introduced cysteine at position V476 (located above the BC) and H483 (located below it) indicated that BC gate opening required movements around the BC point (Holmgren et al., 1998). Electron paramagnetic resonance (EPR) studies of spin-labeled introduced cysteine residues in the vicinity of the BC region in KcsA showed translations and counterclockwise rotations of the M2 helices during BC gate opening. From these distance measurements a gating mechanism was proposed whereby the M2 helices tilt away from the central pore axis and the BC forms an apparatus that from a top view opens and closes like the diaphragm of a camera (Perozo et al., 1999; Liu et al., 2001).

In 2002 the prokaryotic 2Tm-1P Ca^2+^-activated K channel MthK was the first K channel to be crystallized in the open conformation. This structure showed indeed a tilted M2 helix that splays open below the level of a highly conserved glycine residue (G466 in *Shaker*; Jiang et al., 2002a). Comparing this “bent open” MthK structure with KcsA supported a gating mechanism whereby the M2 helices convert from an almost straight α-helical closed conformation to a bent-open one (Jiang et al., 2002b; Figure 2B). Mass tagging experiments in KcsA support this gating model as substantial movements of the intracellular half of the M2 helix were observed during BC gate opening (Kelly and Gross, 2003). An open KcsA model based on the MthK structure suggested that the bottom part of the M2 segment tilts away from the central axis such that the BC point widens up to a 12 Å pore diameter allowing large drug molecules to access the central cavity (Jiang et al., 2002b). Resolving the open state structure of both KcsA (Cuello et al., 2010a,b) and the KirBac3.1 (Bavro et al., 2012) channel showed indeed the bending of the M2 helix whereby the BC widens to a diameter of about 10 Å, which is somewhat smaller than the pore widening in MthK (Liu et al., 2001; Uysal et al., 2011).

While this M2 tilting away from the central pore axis is a very elegant gating mechanism, the question remained whether the BC gate in Kv channels operates in a similar way. The first question is whether the original KcsA and KirBac1.1 structures resemble the closed state of the BC gate in *Shaker*. The finding that the modification of cysteine residues within S6_c_ is protected by application of pore blockers strongly dispelled this idea and suggested that the S6 helix in *Shaker* remains partly bent even in the closed state (del Camino et al., 2000). Second, Cd^2+^ bridging studies of accessible cysteine residues provided distance constraints and suggested that the S6_c_ after the BC point remains closer together in the open conformation than in MthK (Webster et al., 2004). This view was confirmed with the 3D crystal structure of the Kv1.2 channel in the open state, but the BC constriction still widens up to 12 Å in diameter which easily allows access of QA derivatives (8–12 Å in diameter; Long et al., 2005). These results resulted to a bent S6 model of the BC gate in *Shaker* for both the open and closed conformation excluding a mechanism whereby the S6_c_ switched between a straight α-helix and a bent-open situation (del Camino et al., 2000; Webster et al., 2004). Furthermore, this bending of S6 does not occur at G466 but at a conserved PXP motif (P473-X-P475) located further down in S6 (Figure 1; del Camino et al., 2000; Long et al., 2005).

Thus while in all K channels investigated to date, an increase in diameter at the level of the BC explains channel opening, the molecular rearrangements appear different. Whereas BC gate opening in the 2Tm-1P channels appears to involve conversion of the M2 helix from a straight to a bent conformation, the S6 helix in Kv channels remains kinked at the level of a conserved PXP motif resulting in a bent-open S6 conformation in both the open and closed state (Figures 2B,C). One notable exception might be the Kv11.1 (hERG) channel that similar as the 2TM-1P channels lacks these proline residues in S6_c_ making its BC gate structure and gating mechanism different (Cheng and Claydon, 2012). Nevertheless, in both mechanisms of BC gate opening the molecular movements at the S6_c_ are most likely detected by the SF gate. Crystallographic studies of the KcsA channel indicated that the central located S6 residue F103 (which is the counterpart of I470 in *Shaker*) forms a key residue in the molecular coupling between the BC gate and the SF (Cuello et al., 2010a,b). However, KcsA belongs to the 2TM-1P K channels and although the communication appears to traverse mainly along the S6 segment, the molecular coupling between BC gate and SF may be more complicated in Kv channels with a 6Tm-1P topology. Indeed, investigating the communication between BC gate and C-type inactivation at the SF in the Kv11.1 channel showed that in addition to the S6 segment also other channel regions are involved (Wang et al., 2011). Although these findings do not necessarily apply to all types of Kv channels, they highlight that the coupling between BC gate and SF might be more complex.

## Operation of the BC Gate: Requirement of a Hinge Region

Within either gating mechanism, the opening and closure of the BC gate requires a flexible hinge that decouples the pre- and post-hinge portion of the M2 or S6 helix, respectively. In the context of their propensity to break an α-helix, both glycine and proline residues are the obvious candidates to destabilize the helix and to form a hinge that allows tilting or swiveling motions (Tieleman et al., 2001; Bright and Sansom, 2003). The open pore structure of the prokaryotic channels KcsA (Cuello et al., 2010a,b), KirBac3.1 (Bavro et al., 2012), MthK (Jiang et al., 2002a), and KvAP (Jiang et al., 2003) showed that the bending point of the inner pore helix is at a highly conserved glycine residue in the middle of the helix (Guda et al., 2007). Extrapolating this to *Shaker* (and other Kv channels) suggested that glycine residue 466 (Figure 1D) located about eight residues above the BC region would act as the gating hinge (Jiang et al., 2002a,b, 2003; Yifrach and MacKinnon, 2002; Magidovich and Yifrach, 2004). Substituting G466 in *Shaker* by an α-helix promoting alanine residue indeed resulted in non-functional channels that could be rescued by introducing a glycine one position upstream (at residue 465). This highlights the requirement of a glycine in the middle of the S6 segment for voltage-dependent gating in Kv channels (Ding et al., 2005).

Besides this conserved glycine, *Shaker* and other Kv channels contain a tandem proline (PXP) motif seven residues more downstream (Figure 1D) that kinks the S6 helix and creates the bent S6 model. In contrast to KcsA, the KirBac1.1 channel has a glycine residue (G143) at the equivalent position of the second proline residue of the PXP motif and it was proposed to form a pivoting point for gating while the centrally conserved G134 (equivalent of G466 in *Shaker*) might be more important for protein packing (Kuo et al., 2003). However, MD simulations showed that the M2 helix displays bending motions at both glycine positions (Domene et al., 2005; Grottesi et al., 2005). In the *Shaker*-type Kv1.5 channel, functional data underscored the need of S6-helix destabilizing proline residues for voltage-dependent channel gating (Labro et al., 2003) and also in other Kv channels alanine substitutions for the prolines of the PXP motif were not well tolerated (Hackos et al., 2002; Yifrach and MacKinnon, 2002; Harris et al., 2003; Bhattacharji et al., 2006; Seebohm et al., 2006). Furthermore, exchanging the pore module of *Shaker* by its KcsA counterpart that contained the glycine but not the PXP motif resulted in electrically silent channels (Caprini et al., 2001) but a voltage-dependent chimera was obtained when the PXP sequence was re-introduced (Lu et al., 2002; Caprini et al., 2005). These data underscored the importance of this PXP motif for voltage-dependent channel gating which was further supported by the 3D crystal structure of Kv1.2 showing that the S6 helix indeed bends open at this PXP motif (Long et al., 2005). Interestingly, the so-called “silent” Kv channels (that are unable to form functional homotetrameric Kv channels) all lack the second proline of the PXP motif (Figure 1D; Bocksteins and Snyders, 2012).

Does this PXP motif forms a rigid kink or does it function as a flexible hinge? The observation that mutations of this motif affect the channel’s gating kinetics (Hackos et al., 2002; Labro et al., 2003) and that substituting the PXPV sequence in Kv1.5 by AXPP resulted in channels that switched between a fast and a slow activation mode, strongly support the notion that this region reorients during BC gate opening and closure (Labro et al., 2008). In addition to these functional studies, several MD simulations showed an increased flexibility of the S6 helix in the vicinity of the PXP motif and support the formation of a gating hinge at this level (Mashl and Jakobsson, 2008; Imbrici et al., 2009; Denning and Woolf, 2010). These simulations also strengthen the difference in the mechanism of BC gate opening in channels that only possess the central glycine (KcsA/MthK) and the PXP containing Kv channels (Choe and Grabe, 2009).

Metal-bridging studies of the V474C mutant (which is the central residue of the PXP motif) showed that the position of the side chain does not change much with opening suggesting that the residue remains quite stationary during gating (Webster et al., 2004). Although speculative, this suggests that the largest part of the machinery that moves for opening or closing the BC gate is around or downstream of this valine residue. Thus in *Shaker*-type channels the PXP motif would form the main gating hinge or at least creates a second one in addition to the conserved glycine (G466 in *Shaker)* that is flexible in channels like KcsA, MthK, KvAP, and GIRK4 (Jiang et al., 2002a,b, 2003; Jin et al., 2002). In KCNQ1 (Kv7.1) channels the central glycine is missing and the PAG motif (homologous to the *Shaker* PXP motif, Figure 1D) is most important for channel gating (Seebohm et al., 2006). In contrast, its closest relative KCNQ2 does possess the central glycine and mutation to an alanine is not tolerated making firm conclusions based on sequence similarities difficult (Seebohm et al., 2006). Studies in Kir3.4 showed that pivoting does not occur at the glycine itself but one residue upstream (Rosenhouse-Dantsker and Logothetis, 2006). Although glycine and proline are the residues that have the highest intrinsic propensity for destabilizing an α-helix, the effective degree of destabilization and helix rigidity is determined by the overall residue composition of the helix (Rosenhouse-Dantsker and Logothetis, 2006).

In contrast to the other Kv channels, the hERG channel does not contain a PXP motif in the S6_C_ suggesting that its closed state resembles more the straight α-helix KcsA conformation than the bent-open Kv model. However, besides the centrally located glycine, hERG contains also a glycine at the level of the PXP motif (Figure 1D). Remarkably, both glycine residues are not important for voltage-dependent channel gating which led to the idea that the S6 helix of hERG is indeed more rigid than that of other Kv channels (Hardman et al., 2007). Such a difference in rigidity might result in a different mechanistic operation of the BC gate (Cheng and Claydon, 2012). Depending on the preferred conformation of the BC gate, the work performed by the VSD is either to open the gate or to close it. It is quite possible that the preferred (lowest energy) conformational state of the BC gate differs between the different types of Kv channels. For *Shaker* it has been proposed that the BC gate prefers the closed conformation because mutations more often promote the open state than the closed one indicating that the latter is harder to affect and thus represents the intrinsic lower state (Yifrach and MacKinnon, 2002). However, some caution is needed because if the mutation distorts the coupling with the VSD then the opposite is true which might be the scenario in the hERG and KCNQ1 channels (Tristani-Firouzi et al., 2002; Ferrer et al., 2006; Choveau et al., 2011; Labro et al., 2011). Furthermore, the work performed by the VSD might also be dual since both the open and closed conformations of the BC gate represent an energetic minimum. Indeed, it has been shown that BC gate opening stabilizes the VSD in the open conformation (Batulan et al., 2010). Consequently, the VSD needs to pull the BC gate open, but once the gate is fully open the VSD needs to push actively to close it again (Jensen et al., 2012).

In conclusion, K channels contain two gates in series: (1) the BC gate located at the intracellular BC of the inner pore helix (M2 or S6) and (2) the SF gate at the extracellular end of the pore (Figure 1C). Consequently, upon membrane depolarization the K^+^ pore traverses a sequence of events that most likely includes the following conformations (excluding closed state inactivation): (1) BC-closed/SF-intermediate, (2) BC-open/SF-intermediate, (3) BC-open/SF-open, and (4) BC-open/SF-inactivated (Figure 2). In Kv channels, and most likely also the other types of K channels, the BC gate is the main activation gate that is under control of various environmental stimuli such as the membrane potential. From the point of view of the K^+^ ion, the BC gate apparatus displays an iris-like motion at the level where the inner helices cross similar to the opening or closure of the diaphragm from a camera. Two main gating mechanisms have been proposed, in case of the 2TM-1P K channels KcsA and MthK the inner M2 pore helix tilts away from the central K^+^ permeation pathway and converts from an almost straight α-helix conformation (BC gate closed) to a bent one (BC gate open; Figure 2B). In Kv channels the inner S6 pore helix remains bent in both the open and the closed state (bent-open S6 model) displaying most likely more swiveling motions (Figure 2C). The difference between inner pore (BC gate) movements may find its origin in their mechanism of channel opening/closure: BC gate opening in KcsA or MthK relies on pH or Ca^2+^ sensing that involves structural changes in the C-terminally located pH/Ca^2+^ sensing domain (Jiang et al., 2002a; Thompson et al., 2008; Uysal et al., 2009, 2011); in contrast, the BC gate in Kv channels is under direct control of the VSD that is located next to it (Long et al., 2005), and the S6_C_ probably needs to remain bent to maintain contact with the VSD in the closed state (Figure 2D). However, while the VSD directly controls the status of the BC gate, it is not the sole modulator for the overall channel status and conformational changes in NH_2_- and COOH-terminal domains can modulate the gating kinetics (Barros et al., 2012).

## Conflict of Interest Statement

The authors declare that the research was conducted in the absence of any commercial or financial relationships that could be construed as a potential conflict of interest.
